# C-Reactive Protein in Peritoneal Fluid for Predicting Anastomotic Leakage After Colorectal Cancer Surgery: A Systematic Review and Meta-Analysis

**DOI:** 10.3390/jcm14062099

**Published:** 2025-03-19

**Authors:** Tharith Vun, Zhanghao Wu, Chetra Chea, Weidong Liu, Ran Tao, Youming Deng

**Affiliations:** Department of General Surgery, Xiangya Hospital, Central South University, Changsha 410008, China; tharith@csu.edu.cn (T.V.); wuzhh17@csu.edu.cn (Z.W.); 238119005@csu.edu.cn (C.C.); trxyyy@csu.edu.cn (R.T.); dymkingdom@csu.edu.cn (Y.D.)

**Keywords:** anastomotic leakage, peritoneal fluid, C-reactive protein, colorectal cancer surgery

## Abstract

**Background**: Anastomotic leakage (AL) is a serious and potentially fatal complication that can occur after colorectal cancer (CRC) surgery, and it significantly affects patient recovery and increases morbidity. While serum C-reactive protein (CRP) is a recognized systemic inflammatory marker, the level of CRP in peritoneal fluid may serve as a more specific and localized biomarker for early AL detection. This meta-analysis explores the diagnostic potential of peritoneal fluid CRP, aiming to enhance postoperative care for CRC patients. **Methods:** A comprehensive literature search was conducted following the PRISMA guidelines. Eligible studies were included based on strict inclusion and exclusion criteria. Diagnostic accuracy was pooled using a random-effects model. The risk of bias was assessed using the QUADAS-2 tool. **Results:** The pooled sensitivity and specificity were 0.74 and 0.83, respectively, with an area under the curve (AUC) of 0.84, indicating good diagnostic accuracy. The overall diagnostic performance was consistent for sensitivity with no significant heterogeneity, but high heterogeneity was observed for specificity, suggesting variability between studies. Subgroup analysis revealed improved diagnostic performance between postoperative days 5–7 and higher CRP cut-off values (70–150 mg/L). The analysis confirmed the stability of the results through a sensitivity analysis and found no significant publication bias. **Conclusions:** Peritoneal fluid CRP is a reliable biomarker for detecting AL after CRC surgery, especially in the later postoperative period. However, heterogeneity in study methodologies and patient populations limits the generalizability of the findings. Future research should focus on standardizing protocols and exploring additional biomarkers to improve diagnostic accuracy.

## 1. Introduction

Colorectal cancer (CRC) is the third most commonly diagnosed cancer and the second leading cause of cancer-related mortality worldwide, accounting for approximately 10% of global cancer cases and deaths [1]. The incidence of CRC varies significantly across regions, with the highest rates being observed in developed countries, which is likely due to dietary and lifestyle factors [2]. Despite advancements in screening and treatment, the prognosis of CRC remains closely tied to the stage at diagnosis, emphasizing the importance of early detection for favorable outcomes [3].

Surgical resection remains the cornerstone of treatment for localized CRC but is often accompanied by substantial postoperative complications that negatively impact patient outcomes and quality of life. Acute postoperative complications include bleeding, infection, bowel obstruction, and anastomotic leakage (AL) [4]. Among these, AL is particularly concerning due to its severe clinical consequences [5].

AL is defined as a defect in the surgical joint between two segments of the intestine, leading to the leakage of luminal contents into the peritoneal cavity [6]. The pathophysiology of AL involves multiple factors, including surgical techniques, local blood supply, tissue integrity, and the patient’s immune response [7]. AL typically presents within the first week post-surgery [8], with symptoms such as abdominal pain, fever, tachycardia, reduced or absent bowel sounds, abdominal distension, peritoneal signs (rebound tenderness, guarding), and signs of sepsis. Changes in drainage fluid (cloudiness, purulence, fecal odor) and increased output may further indicate leakage [9,10]. Recognizing these early symptoms and signs is crucial for timely diagnosis and intervention, reducing the risk of severe complications.

The complications of AL are severe, and they include peritonitis, sepsis, abscess formation, prolonged hospitalization, and increased mortality rates [11]. Early detection of AL is essential for timely intervention, which can significantly reduce morbidity and improve survival and overall outcomes [12].

Leakage from the anastomotic site results in the escape of intestinal contents into the peritoneal cavity, triggering a robust inflammatory response. This response is characterized by the recruitment of immune cells and the release of inflammatory cytokines such as IL-6 and TNF-α, which stimulate the production of C-reactive protein (CRP). CRP is an acute-phase reactant protein synthesized in the liver, and it is widely recognized for its role in systemic inflammation. Elevated CRP levels are commonly associated with postoperative complications, including infection and tissue damage. Increased permeability of the peritoneum allows inflammatory mediators to enter the peritoneal fluid. CRP binds to damaged cells and pathogens, activates the complement system, and promotes phagocytosis by immune cells. In the context of anastomotic leakage, CRP serves as a sensitive marker for detecting leakage and monitoring the severity of inflammatory response. Elevated CRP levels in the peritoneal fluid (drainage fluid) provide valuable insights into the local inflammatory response at the leak [13,14,15,16,17,18].

Moreover, recent research has explored the potential of CRP levels in the peritoneal fluid as a more specific marker for detecting local inflammation related to anastomotic leakage. As the peritoneal fluid directly interfaces with the surgical site, its CRP concentration may provide early evidence of local inflammatory changes, potentially serving as a sensitive and localized predictor of leakage [13,19,20,21].

Despite multiple studies exploring the role of CRP levels in peritoneal fluid, there is significant variability in their findings, with inconsistent results obtained across different clinical settings. To date, no comprehensive meta-analysis has systematically consolidated the evidence to clarify its diagnostic value. This study addresses the gap by providing pooled estimates of diagnostic accuracy, analyzing heterogeneity, and performing subgroup analysis to determine the optimal postoperative window and cut-off values for CRP measurement and provide guidance for its potential application in clinical practice.

## 2. Materials and Methods

### 2.1. Information Sources

This review was conducted in accordance with the Preferred Reporting Items for Systematic Reviews and Meta-Analyses of Diagnostic Test Accuracy (PRISMA-DTA) Statement and adhered to the PRISMA 2020 guidelines [22]. The study protocol was registered in the PROSPERO database (ID: CRD42024621595). We performed a comprehensive and systematic search of multiple databases to identify relevant studies published through November 2024. The databases searched included PubMed, Web of Science, Embase, Cochrane Library, Wanfang (www.wanfangdata.com.cn/index.html (accessed on 31 November 2024), and the China National Knowledge Infrastructure (CNKI) (www.cnki.net). We utilized a combination of Medical Subject Headings (MeSH) and free-text terms related to “C-reactive protein”, “peritoneal fluid”, “colorectal cancer”, “surgery”, and “anastomotic leakage.” The search terms were combined using Boolean operators (AND, OR) with a query structured as follows: ((c-reactive protein) AND (anastomotic leak) AND (peritoneal fluid)) AND ((predict) OR (colorectal cancer surgery)). In addition, we manually reviewed the reference lists of the included studies and relevant review articles to ensure that no relevant studies were overlooked.

### 2.2. Eligibility Criteria

To ensure the relevance and quality of the studies included in our meta-analysis, we established strict inclusion and exclusion criteria. The inclusion criteria were the following: (1) patients undergoing colorectal cancer surgery, (2) assessment of CRP levels in peritoneal fluid post-surgery, (3) reported outcomes related to anastomotic leakage, and (4) study designs, including prospective, retrospective, and case–control methodologies. The exclusion criteria were the following: (1) case reports, reviews, editorials, or other publications lacking primary data, and (2) studies that did not provide key diagnostic performance data for extraction (e.g., sensitivity, specificity, number of AL patients), or studies with an AUC below 0.7.

### 2.3. Publication Screening and Data Extraction

Two independent reviewers conducted the literature screening. First, titles and abstracts were screened to exclude irrelevant studies. Then, the full-text articles of the remaining studies were assessed based on the predefined inclusion and exclusion criteria to determine eligibility. The reasons for exclusion were systematically documented. Data were extracted from the included studies using a standardized data extraction form. Any discrepancies between the reviewers were resolved through discussion or consultation with a third reviewer. The extracted data included the following variables: authors, year of publication, country, study design, total number of patients, number of AL patients, postoperative day (POD), cut-off value, area under the curve (AUC), sensitivity, specificity, methodology, inclusion and exclusion criteria, study limitations, clinical implications, and comments.

### 2.4. Quality Assessment

The Quality Assessment of Diagnostic Accuracy Studies 2 (QUADAS-2) tool was applied using the STATA/SE software (Version 15.1) software. Four key components were evaluated: patient selection, index test, reference standard, and flow and timing. The risk of bias was categorized as “low”, “high”, or “unclear” based on responses of “yes”, “no”, or “unclear” to specific questions in each section. A “yes” response to all questions within a component indicated a low risk of bias, whereas a “no” response indicated a high risk. An “unclear” rating was assigned when insufficient information was provided. Any conflicts in the quality assessment process were resolved through discussion with a fourth reviewer.

### 2.5. Synthesis Methods

The pooled estimates of sensitivity (SEN), specificity (SPE), positive likelihood ratio (PLR), negative likelihood ratio (NLR), diagnostic odds ratio (DOR), and area under the curve (AUC) were calculated using a random-effects model to account for variability among studies. Summary receiver operating characteristic (SROC) curves were generated to summarize the overall diagnostic accuracy, with the AUC quantifying the diagnostic performance. Heterogeneity was assessed using the I^2^ statistic and Cochran’s Q test (*p*-values), with an I^2^ value greater than 50% indicating substantial heterogeneity. A sensitivity analysis was performed to assess the influence of individual studies on the overall pooled estimates. Publication bias was assessed using Deeks’ funnel plot asymmetry test, with a *p*-value of less than 0.10 being considered indicative of significant publication bias. Subgroup analyses (POD 3–5, POD 5–7, CV 40–70, CV 70–150) were conducted to compare diagnostic accuracy at different time points and levels of CRP. This meta-analysis adhered to robust statistical methods using the Stata/SE software (version 15.1), ensuring reliable pooled estimates.

## 3. Results

### 3.1. Study Selection

**Figure 1:**
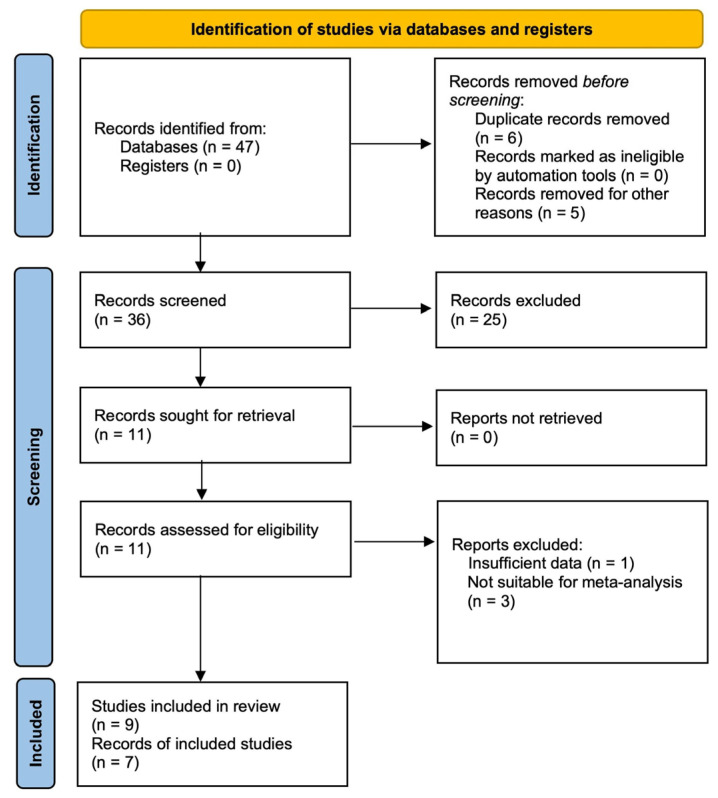
PRISMA flow diagram.

Of the 47 records initially identified in various databases, 11 full-text articles were assessed for eligibility after excluding 36 records due to duplication, irrelevance to the topic, or classification as review or meta-analysis articles. Ultimately, seven articles, comprising nine individual studies, were included in the qualitative synthesis. Four articles were excluded due to their insufficient data or unsuitability for meta-analysis (Figure 1).

### 3.2. Study Characteristics

Table 1 provides a comparative overview of the included studies, detailing the main author, year of publication, country, study design, type of resection, measurement techniques, and study period. It also presents various CRP cut-off values measured on different PODs (days 3–7) along with the diagnostic performance metrics, including sensitivity, specificity, and AUC, to evaluate the effectiveness of each study in predicting AL.

Table 2 offers a comprehensive analysis of all included studies and subgroup analyses based on the postoperative day (POD 3–5, POD 5–7) and CRP cut-off values (CV 40–70 mg/L and CV 70–150 mg/L). This table includes information on heterogeneity, reporting the I^2^ statistic and *p*-value to assess variability across studies. The inclusion of this information facilitates a comparison between the overall analysis and the analysis of specific postoperative subgroups.

Table 3 outlines the inclusion and exclusion criteria for each study, providing details on patient selection and study limitations. It underscores the clinical relevance of monitoring specific biomarkers for the early detection of AL and identifies potential areas for future research to enhance predictive accuracy.

Table 4 stratifies the severity of AL based on the POD, CRP levels, and clinical presentation, offering practical recommendations for AL management.

**Figure 2:**
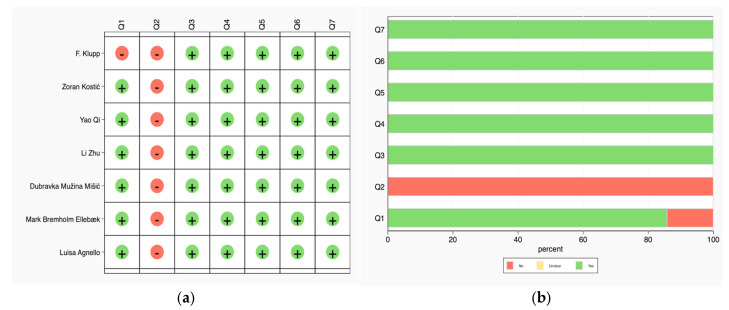
(**a**) Risk of bias (Q1 = patient selection, Q2 = index test, Q3 = reference standard, Q4 = flow timing). (**b**) Applicability concerns (Q5 = patient selection, Q6 = index test, Q7 = reference standard).

A graph and summary of the risk of bias and applicability concerns are shown in Figure 2a,b. Most of the studies were considered to have a low risk of bias in most domains except for the index test (Q2), which consistently showed a high risk of bias across all studies. Applicability concerns were generally low, with consistent “yes” scores across Q5 to Q7. These results suggest that, while patient selection, reference standards, and flow timing are generally reliable, the index test used in these studies poses a significant risk of bias. This should be considered when interpreting the findings of these studies.

### 3.3. Results of Syntheses

**Figure 3:**
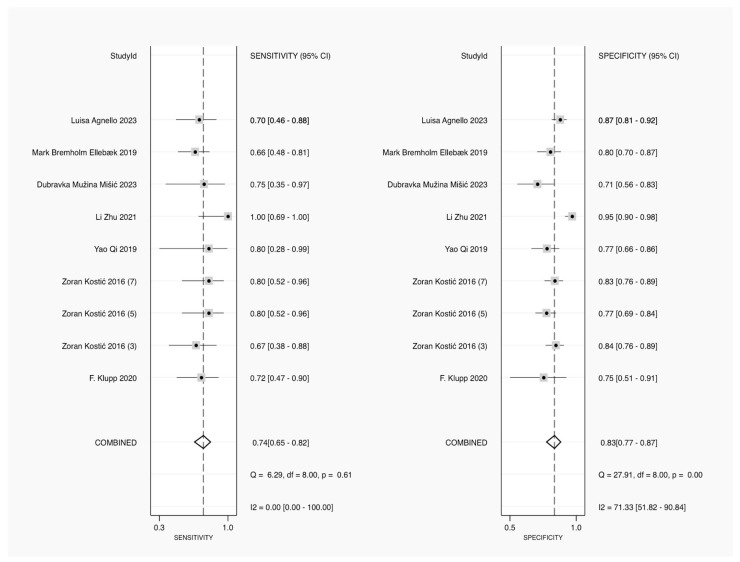
Forest plot of pooled sensitivity and specificity for CRP in diagnosing AL [23,24,25,26,27,28,29].

Sensitivity estimates were displayed for each study, representing the test’s ability to correctly identify patients with AL. The combined sensitivity, or pooled result, was 0.74 (95% CI: 0.65–0.82), with no significant heterogeneity, as indicated by an I^2^ statistic of 0.00 and a Q-statistic of 6.29 (*p* = 0.61). This finding suggests consistency across studies on sensitivity. Specificity estimates are shown, reflecting the ability of the test to correctly identify patients without AL. The combined specificity was 0.83 (95% CI: 0.77–0.87), but the I^2^ statistic for heterogeneity was 71.33, indicating substantial variability across the studies. The Q-statistic for specificity was 27.91 (*p* = 0.00), confirming significant heterogeneity. Overall, while the pooled sensitivity of CRP was moderately high and consistent across studies, the specificity showed greater variability, suggesting differences in CRP’s effectiveness in ruling out leakage in different settings (Figure 3).

**Figure 4:**
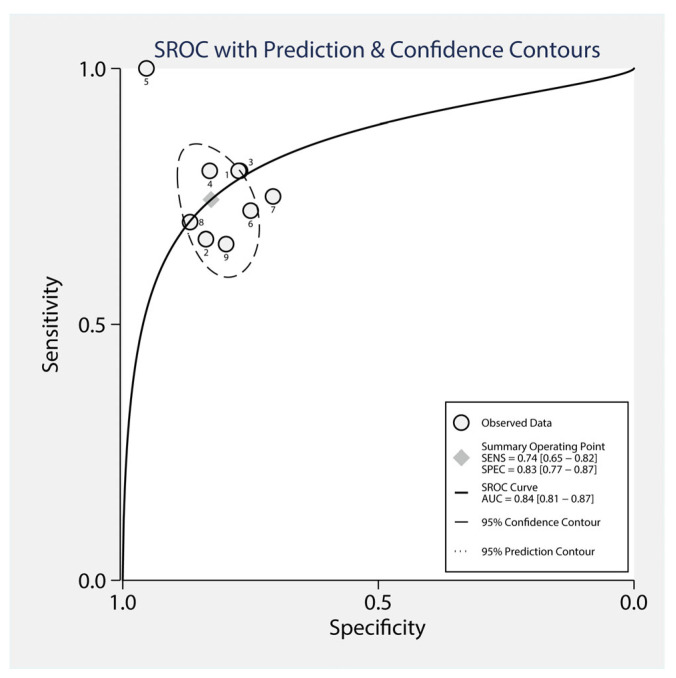
Summary receiver operating characteristic (SROC) curve for the diagnostic accuracy of CRP in diagnosing AL.

The summary operating point is shown as a gray diamond in Figure 4, representing a pooled sensitivity of 0.74 (95% CI: 0.65–0.82) and a pooled specificity of 0.83 (95% CI: 0.77–0.87). The area under the curve (AUC) for the SROC was 0.84 (95% CI: 0.81–0.87), indicating good overall diagnostic accuracy of CRP in detecting anastomotic leakage. The solid black line represents the SROC curve, and the dashed lines indicate the 95% confidence contour (indicating the uncertainty around the summary operating point) and the 95% prediction contour (representing where future studies are expected to fall). The observed data points from the individual studies are shown as circles distributed around the SROC curve (Figure 4).

**Figure 5:**
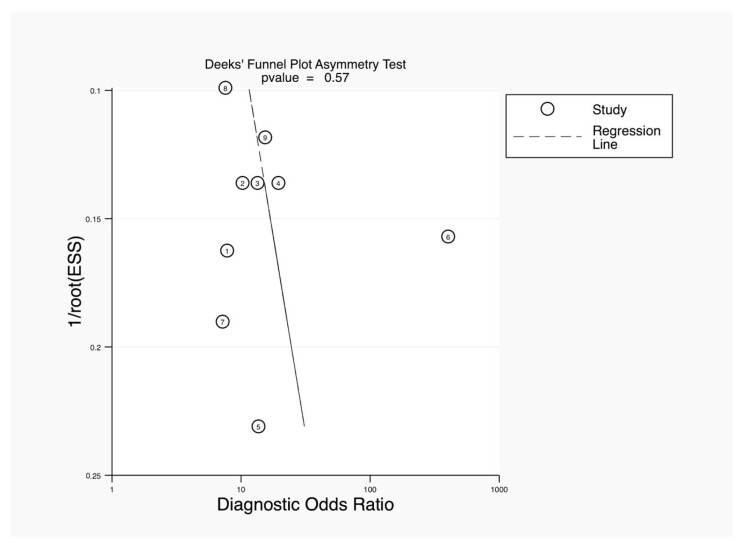
Deeks’ funnel plot asymmetry test.

The Deeks’ funnel plot asymmetry test was used to assess publication bias in a meta-analysis of diagnostic accuracy studies. The *p*-value for the asymmetry test was 0.57, which is well above the common threshold of 0.10. This indicated that there was no significant evidence of publication bias in the studies included in this analysis. The absence of asymmetry in the funnel plot further supports this conclusion, as a symmetrical plot is typically expected when publication bias is absent (Figure 5).

**Figure 6:**
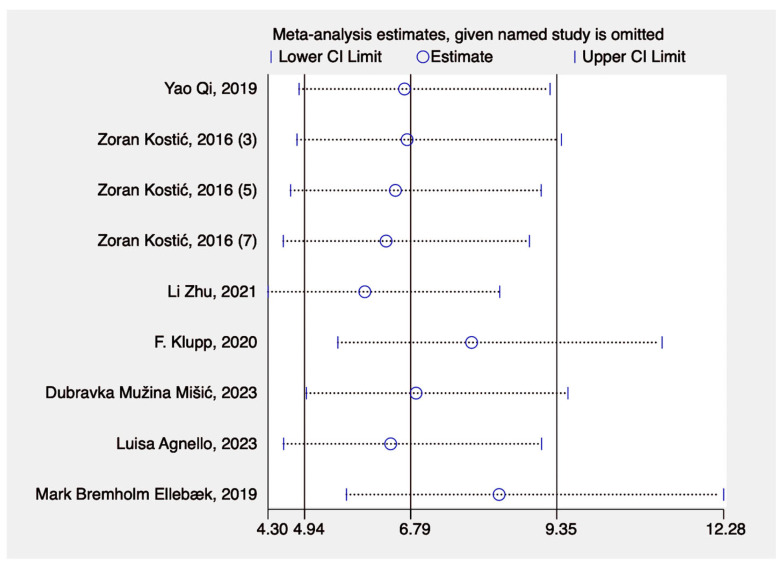
Sensitivity analysis [23,24,25,26,27,28,29].

The leave-one-out sensitivity analysis showed the estimates of the meta-analysis (with their 95% confidence intervals) when each study was omitted one at a time. The x-axis displays the effect size estimate (ranging from 4.30 to 12.28), while the y-axis lists individual studies by author and year. Each row shows how the pooled estimate would change if a particular study were excluded. The circles represent the new estimate of the pooled effect, and the horizontal lines indicate the lower and upper confidence limits, respectively. The consistent placement of these estimates and confidence intervals across studies suggests that omitting any single study does not significantly affect the overall pooled effect size. This indicates that the meta-analysis results are robust and not overly dependent on any study (Figure 6).

In the overall analysis of nine studies, the pooled SEN was 0.74 (95% CI: 0.65–0.82), and the SPE was 0.83 (95% CI: 0.77–0.87), indicating moderately high diagnostic accuracy. The PLR was 4.3 (95% CI: 3.0–6.1), and the NLR was 0.31 (95% CI: 0.22–0.44). The DOR was 14 (95% CI: 7–27), and the AUC was 0.84 (95% CI: 0.81–0.87), showing strong diagnostic performance. There was no significant heterogeneity (I^2^ = 0, *p* = 0.433).

In the POD 3–5 subgroup (seven studies), the SEN was slightly higher at 0.76 (95% CI: 0.65–0.85), and the SPE was 0.83 (95% CI: 0.75–0.89). The PLR was 4.5 (95% CI: 2.8–7.1), and the NLR was 0.29 (95% CI: 0.18–0.45). The DOR was 16 (95% CI: 7–36), and the AUC was 0.85 (95% CI: 0.82–0.88). There was no significant heterogeneity (I^2^ = 0, *p* = 0.395).

In the POD 5–7 subgroup (five studies), the SEN increased to 0.82 (95% CI: 0.59–0.94), and the SPE rose slightly to 0.84 (95% CI: 0.75–0.90). The PLR was 5.1 (95% CI: 2.7–9.5), and the NLR was 0.21 (95% CI: 0.08–0.59). The DOR was 24 (95% CI: 5–117), and the AUC was the highest at 0.90 (95% CI: 0.87–0.92). There was no significant heterogeneity (I^2^ = 0, *p* = 0.421), indicating a stable result.

We found that for most studies included in the review, the cut-off values (CVs) increased over time, ranging from 42 mg/L to 147 mg/L. Therefore, we divided the CVs into two subgroups: CVs of 40–70 mg/L and CVs of 70–150 mg/L. Making this division at 70 mg/L allowed for a balanced categorization of studies across both subgroups, and these intervals aligned with common clinical thresholds used in other studies. This ensured that the studies in each category were comparable in number, facilitating better subgroup analysis when evaluating diagnostic performance. In the CV 40–70 mg/L subgroup (five studies), the SEN was 0.77 (95% CI: 0.65–0.86), and the SPE dropped to 0.78 (95% CI: 0.74–0.82). The PLR was 3.5 (95% CI: 2.8–4.4), and the NLR was 0.29 (95% CI: 0.18–0.47). The DOR was 12 (95% CI: 6–23), and the AUC was 0.84 (95% CI: 0.81–0.87). However, the heterogeneity was significant (I^2^ = 100, *p* = 0.500), indicating variability among the studies in this subgroup.

In the CV 70–150 mg/L subgroup (four studies), the SEN was 0.77 (95% CI: 0.55–0.91), and the SPE improved to 0.88 (95% CI: 0.80–0.93). The PLR was 6.3 (95% CI: 3.1–12.8), and the NLR was 0.26 (95% CI: 0.11–0.61). The DOR was 25 (95% CI: 5–112), and the AUC was 0.91 (95% CI: 0.88–0.93). The heterogeneity was not significant (I^2^ = 0, *p* = 0.449) (Table 2).

## 4. Discussion

This meta-analysis demonstrated that peritoneal fluid CRP is a promising biomarker for detecting AL after colorectal cancer surgery, exhibiting moderate to high diagnostic accuracy. The pooled sensitivity of 0.74 suggests that peritoneal fluid CRP effectively identifies patients with AL, a finding consistently observed across studies, as indicated by the absence of heterogeneity. This reinforces its reliability and potential clinical applicability. Conversely, the pooled specificity of 0.83 reflects the ability of CRP to correctly exclude AL in postoperative patients. However, the high heterogeneity in specificity suggests significant variability across studies, which is likely due to differences in CRP measurement techniques, cut-off values, and patient populations (Table 3).

The SROC analysis demonstrated an AUC of 0.84, confirming that peritoneal fluid CRP is a reliable diagnostic marker for AL. This performance aligns with other established inflammatory biomarkers, supporting its potential integration into routine postoperative monitoring. While the strong sensitivity and high AUC suggest that peritoneal fluid CRP is a valuable tool for early AL detection, the significant heterogeneity in specificity underscores the need for standardization of CRP measurement protocols across different clinical settings to enhance diagnostic reliability and comparability.

The absence of significant publication bias enhances the credibility of our findings. Additionally, the leave-one-out sensitivity analysis demonstrated the robustness of the findings, as omitting any single study did not significantly affect the pooled estimates. This underscores the stability and generalizability of the conclusions drawn from this meta-analysis.

The subgroup analyses provided further insights into how the diagnostic accuracy of CRP depends on the postoperative day of measurement and the CRP cut-off values employed. In the POD 5–7 subgroup, CRP demonstrated higher sensitivity (0.82) and an AUC of 0.90, suggesting that CRP is more accurate for detecting AL in the later postoperative period. This aligns with the hypothesis that CRP levels rise over time as the inflammatory response intensifies, making it a more effective biomarker for POD 5–7. Conversely, in the POD 3–5 subgroup, the sensitivity was slightly lower at 0.76, and the AUC was 0.85, indicating that while CRP was useful for earlier detection of AL postoperatively, its diagnostic accuracy improved with time. This could be attributed to the fact that AL may not be fully developed by POD 3–5 or that the inflammatory response is still in its early stages.

Regarding CRP cut-off values, the CV 70–150 mg/L subgroup showed the highest diagnostic performance, with a DOR of 25 and an AUC of 0.91. This suggests that higher CRP cut-off values offer better specificity (0.88) and diagnostic accuracy, allowing clinicians to rule out patients without AL. In contrast, the CV 40–70 mg/L subgroup demonstrated significant heterogeneity (I^2^ = 100%) and lower specificity (0.78), suggesting that lower cut-off values may introduce variability and reduce the diagnostic reliability of this biomarker.

The results of this meta-analysis support the integration of CRP monitoring into the postoperative care of patients undergoing CRC surgery. The strong diagnostic performance of CRP, particularly in the POD 5–7 window and when using higher cut-off values, suggests that it can serve as an effective tool for the early detection of AL. The early detection of AL allows for timely intervention, potentially reducing postoperative morbidity and improving overall outcomes.

In clinical practice, these findings suggest a cautious approach during the early postoperative period. In the CV 40–70 subgroup at POD 3–5, CRP shows moderate diagnostic power, but there is a risk of overdiagnosis due to early inflammation. Clinicians should avoid hasty invasive interventions, such as reoperation or early drainage, without corroborating evidence. It is advisable to combine CRP levels with clinical signs, such as persistent fever, abdominal pain, or abnormal vital signs (e.g., tachycardia, hypotension), to determine whether further investigation is warranted. Continuous monitoring of CRP levels, vital signs, clinical status, complete blood count, and antibiotic management over the following days is essential. A declining CRP trend is reassuring, while a continued rise may necessitate a more aggressive evaluation for AL.

In the CV 40–70 subgroup at POD 5–7, two scenarios are possible if the CRP cut-off value remains the same as during POD 3–5. First, if there are no clinical signs, abnormal vital signs, or leukocytosis, then the elevated CRP level could be due to other causes, such as surgical trauma, and not necessarily AL, indicating the patient can be safely discharged. Second, if clinical signs, abnormal vital signs, or leukocytosis are present, then a minor leak may be suspected. In this case, delayed discharge, conservative treatment (e.g., antibiotics, supportive care), and continued monitoring of CRP are recommended, although CT imaging might not be necessary. If the CRP levels stabilize and the patient’s symptoms improve, them this indicates the condition is under control, and discharge after a few more days of observation is appropriate.

In the CV 70–150 subgroup at POD 3–5, the higher CRP cut-off offers improved specificity, making it a more reliable marker for diagnosing AL. The presence of clinical signs, leukocytosis, or abnormal vital signs in these subgroups increases suspicion of AL. Clinicians should act swiftly to rule out AL, with a lower threshold for initiating diagnostic tests. Contrast-enhanced CT imaging should be promptly considered, as it provides reliable visualization of the anastomotic site and surrounding tissues. However, in the absence of clinical signs, leukocytosis, or abnormal vital signs, continued observation and conservative treatment are more appropriate because the CRP levels increase during this period.

Later in the postoperative course, CRP becomes even more useful. The CV 70–150 subgroup at POD 5–7 demonstrates excellent diagnostic performance. At this stage, AL is likely more advanced, and prompt intervention is crucial to prevent severe complications, such as peritonitis or sepsis. If imaging confirms fluid collections, abscesses, or signs of leakage, collaboration among surgeons, radiologists, and critical care specialists is essential to determine whether conservative management is sufficient or if surgery [30] (e.g., percutaneous drainage, re-anastomosis, stoma) is required. Monitoring CRP levels during drainage procedures also reflects the effectiveness of treatment. For elderly patients, quickly ruling out AL with contrast-enhanced CT and delaying discharge with conservative treatment may still be necessary, even in the absence of clinical signs, leukocytosis, or abnormal vital signs. A declining CRP level after treatment is a positive sign, while continued elevation may indicate ongoing issues, such as incomplete drainage or unresolved infection (Table 4).

In addition to monitoring CRP levels in the peritoneal fluid, comparing CRP levels between serum and peritoneal fluid can provide further diagnostic value. The differential CRP levels in these two biological compartments may reflect localized versus systemic inflammatory responses, offering clinicians a more nuanced view of the patient’s condition [24]. Previous studies have reported that serum CRP levels typically peak around POD 3–5, with values often exceeding 144–172 mg/L in cases of AL [31]. However, due to systemic influences such as other infections or surgical trauma, serum CRP alone has lower specificity for AL detection compared with peritoneal fluid CRP [32]. In contrast, peritoneal fluid CRP, with reported cut-off values ranging from 70–150 mg/L in our study, provides a more direct reflection of localized inflammation, thereby improving diagnostic accuracy. For instance, an elevated CRP level in peritoneal fluid relative to serum may suggest localized inflammation due to AL, whereas comparable levels in both compartments may indicate a more systemic inflammatory response. By analyzing CRP levels in both serum and peritoneal fluid, clinicians can enhance diagnostic accuracy and make more informed decisions regarding the presence and severity of anastomotic leakage [26]. This dual-compartment approach adds another dimension to diagnostic precision, potentially reducing false positives and improving patient management.

Other biomarkers present in peritoneal fluid can further enhance the diagnostic accuracy of AL. Immune markers such as IL-1, IL-6, IL-10, TNF-α, and CCL8, which can be detected on POD 1–2, provide valuable insights into the inflammatory response [25,33,34,35,36,37,38,39,40,41], while ischemia markers such as lactate and pH offer critical information on tissue perfusion and metabolic changes at the anastomotic site [27,42,43,44]. Furthermore, microbiological markers, such as the detection of specific pathogens, can signal bacterial contamination, which is a key indicator of leakage [45,46,47]. By simultaneously measuring and analyzing these diverse biomarkers, clinicians can adopt a more comprehensive diagnostic approach, combining inflammatory, ischemic, and microbial profiles to improve the early detection of AL. The integration of multiple biomarkers can help overcome the limitations of relying on CRP alone, particularly in cases in which CRP may not provide definitive results.

Measuring CRP in peritoneal fluid is a quick, convenient, and painless procedure that effectively monitors local inflammation, helps diagnose complications early, and guides treatment decisions. Despite the strengths of this study, some limitations must be acknowledged. The significant heterogeneity in specificity underscores the variability in CRP cut-off values and measurement techniques used across studies. Additionally, the lack of data on physical examination findings, vital signs, and leukocyte values limits the ability to fully contextualize CRP results. Dividing the CVs into two subgroups may offer some balance in terms of study numbers but introduces challenges related to arbitrary cut-off selection, heterogeneity, overlapping diagnostic performance, and clinical interpretation. Future research should focus on establishing standardized CRP protocols and exploring their use in combination with other biomarkers to improve AL detection. Larger prospective studies are also needed to confirm these findings and determine the optimal CRP cut-off values for use in clinical practice.

## 5. Conclusions

This meta-analysis demonstrated the diagnostic value of peritoneal CRP for detecting AL after CRC surgery. CRP shows high diagnostic accuracy, particularly when measured between POD 5 and 7 and at higher cut-off values. Monitoring trends in CRP can help detect complications early and guide interventions, improving postoperative outcomes. Future studies should refine CRP protocols and explore combinations with other biomarkers. A limitation of this meta-analysis is the variability in study protocols, particularly in CRP measurement techniques and cut-off values, leading to significant heterogeneity in specificity. Additionally, the lack of data on physical examination findings, vital signs, and leukocyte values limits the ability to fully contextualize the CRP results. Standardized measurement methods and larger prospective studies are essential to validate and optimize these findings.

## Figures and Tables

**Table 1:** Summary of studies analyzing C-reactive protein for predicting anastomotic leakage.

Author	Country	Study Design	Resection	SP	P	AL	POD	CV	AUC	SEN	SPE
YaoQi, 2019 [23]	China	Prospective	Rectal	5	80	5	5	57.68	0.855	88	77
Li Zhu, 2021 [24]	China	Retrospective	Colorectal	5	140	10	5	147	0.984	100	95
F.Klupp, 2020 [25]	Germany	Prospective	Colorectal	3	38	18	3	62.92	0.703	72.2	75
Dubravka Muzina Misic, 2023 [26]	Croatia	Prospective	Colorectal	4	59	8	4	55.2	0.833	71	71
Mark Bremholm Ellebæk, 2019 [27]	Denmark	Prospective	Rectal	7	129	35	7	146	0.8	67	80
Luisa-Agnello, 2023 [28]	Italy	Prospective	Colorectal	5	187	20	3	76	0.752	72	87
Zoran Kostic, 2016 [29]	Serbia	Prospective	Colorectal	7	150	15	3	77	0.745	67	84
							5	53	0.879	80	77
							7	42	0.824	80	83

SP: study period (days), P: patient, AL: anastomotic leakage, POD: postoperative day, CV: cut-off value (mg/L), AUC: area under the curve, SEN: sensitivity (%), SPE: specificity (%).

**Table 2:** Meta-analysis of diagnostic accuracy for C-reactive protein in predicting anastomotic leakage: overall and subgroup analyses.

Categories	N	SEN	SPE	PLR	NLR	DOR	AUC	H	
		(95%CI)	(95%CI)	(95%CI)	(95%CI)	(95%CI)	(95%CI)	I^2^	*p*-Value
Overall	9	0.74 (0.65–0.82)	0.83 (0.77–0.87)	4.3 (3–6.1)	0.31 (0.22–0.44)	14 (7–27)	0.84 (0.81–0.87)	0	0.433
Subgroup									
POD 3–5	7	0.76 (0.65–0.85)	0.83 (0.75–0.89)	4.5 (2.8–7.1)	0.29 (0.18–0.45)	16 (7–36)	0.85 (0.82–0.88)	0	0.395
POD 5–7	5	0.82 (0.59–0.94)	0.84 (0.75–0.90)	5.1 (2.7–9.5)	0.21 (0.08–0.59)	24 (5–117)	0.90 (0.87–0.92)	0	0.421
CV 40–70	5	0.77 (0.65–0.86)	0.78 (0.74–0.82)	3.5 (2.8–4.4)	0.29 (0.18–0.47)	12 (6–23)	0.84 (0.81–0.87)	100	0.500
CV 70–150	4	0.77 (0.55–0.91)	0.88 (0.80–0.93)	6.3 (3.1 -12.8)	0.26 (0.11–0.61)	25 (5–112)	0.91 (0.88–0.93)	0	0.449

N: number of studies, SEN: sensitivity, SPE: specificity, PLR: positive likelihood ratio, NLR: negative likelihood ratio, DOR: diagnostic odds ratio, AUC: area under the curve, H: heterogeneity, CI: clearance interval, POD: postoperative day, CV: cut-off value (mg/L).

**Table 3:** Characteristics of Studies Investigating C-reactive protein as a Biomarker for Predicting Anastomotic leakage: Methodology, Inclusion, Exclusion, Limitations, and Clinical Implications.

Author	Methodology *	Inclusion	Exclusion	Limitation	Clinical Implication	Comment
Yao Qi 2019 [23]	Immunoscattering turbidimetry: Peritoneal drainage fluid collected on POD 0, 3, 5.	Laparoscopic Dixon radical resection of rectal cancer from July 2017 to July 2018; Confirmed rectal cancer by preoperative examination and postoperative pathology; No infection before or after surgery.	Severe liver and kidney dysfunction; Other primary malignant tumors; Severe postoperative infection; Crohn’s disease.	No comparison between peritoneal fluid and serum markers for predicting leakage; Small sample size.	Monitoring CRP in drainage fluid post-surgery can guide AL prevention and management.	Future studies should compare peritoneal CRP and PCT with serum markers for AL prediction.
Li Zhu 2021 [24]	Immunoturbidimetry (Beckman Coulter automated biochemical analyzer, Brea, CA, USA): Peritoneal drainage fluid collected on POD 1, 3, 5.	Complete clinical data; Confirmed CRC by colonoscopy and biopsy; Indication for laparoscopic radical surgery; No preoperative infection; No residual tumors confirmed by postoperative pathology.	Autoimmune diseases; Recent use of immunosuppressants or hormones; Severe multi-organs disfunction; Other malignancies; Colorectal tumors with local inflammation and elevated preoperative CRP.	Selection bias, small sample size, retrospective nature.	Dynamic CRP monitoring in serum and drainage fluid aids early AL detection on specific postoperative days.	Validate findings in larger studies; investigate optimal CRP cut-off values in serum and drainage fluid.
F. Klupp 2020 [25]	ELISA (Luminex^®^-based multiplex assay, Austin, TX, USA): Peritoneal drainage fluid collected on POD 1 to 3.	Elective surgery of CRC with a complete set of samples and without other postoperative complications.	Secondary carcinomas; Drainage removal before the POD3; Postoperative infections.	Small sample size and potential confounding factors; limited applicability.	Monitoring CRP in serum and peritoneal fluid on day 3 aids early AL detection, improving outcomes.	Use CRP in peritoneal fluid to monitor local inflammation and AL; validate findings in larger cohorts.
Dubravka Mužina Mišić 2020 [26]	(Latex immunoturbidimetry): Peritoneal drainage fluid collected on POD 1 to 4.	CRC resection with primary anastomosis between January 2019 and October 2019.	Under 18 years; Receiving neoadjuvant CRT; Emergency surgery; On steroid therapy.	Study’s small sample size affects generalizability; Only included patients without other complications.	Monitoring CRP in serum and peritoneal fluid with repetitive day 4 measurements helps early AL detection.	Validate findings with larger cohorts; refine CRP cut-off values; explore CRP’s utility in combination with serum levels.
Mark Bremholm Ellebæk 2019 [27]	Immunoturbidimetry: Peritoneal drainage fluid collected on POD 1 to 7.	Age ≥ 18 years; Biopsy-proven adenocarcinoma of the rectum (lower border within 15 cm from the anal verge by rigid proctoscopy); Potentially curative surgery, irrespective of neoadjuvant therapy; Written informed consent.	Patients not meeting the inclusion criteria were excluded.	Small sample size, single-center design, and need for larger studies noted; logistical challenges affected study duration.	CRP is valuable for early AL detection post-surgery; it complements intraperitoneal lactate in surveillance.	Explore systematic CRP use with other diagnostic tools for AL; CRP can improve early detection and management.
Luisa Agnello 2023 [28]	Immunoscattering turbidimetry (Cobas 8000, Roche Diagnostics, Indianapolis, IN, USA): Peritoneal drainage fluid collected on POD 3, 5.	Aged > 16 years undergoing elective or emergency CRC surgery; Diverticular disease; Inflammatory bowel disease; Reversal of Hartmann’s procedure.	Age < 16 years, no informed consent, no anastomosis performed during the surgical procedure.	Single-center study and small sample size limit generalizability; Larger, multicenter studies needed.	Monitoring LDH, CRP and NLR in drainage fluid allows earlier AL detection and intervention, reducing morbidity.	Validate LDH, CRP and NLR as early AL biomarkers in larger, multicenter studies; explore other potential biomarkers.
Zoran Kostic 2016 [29]	Immunonephelometry autoanalyzer (DADE Behring BN II, Malvern, PA, USA): Peritoneal drainage fluid collected on POD 1, 3, 5, 7.	CRC resection and primary anastomosis.	Clinical signs of infection or inflammatory conditions preoperatively; Tumor recurrence surgery; No adequate postoperative drainage fluid samples.	CRP, as an inflammation marker, should be considered in context; limited reliability as an infection indicator.	CRP measurement in drainage fluid can detect AL early, allowing timely interventions.	Confirm findings and investigate if combining CRP with other biomarkers enhances AL detection accuracy.

*: Peritoneal fluid samples were collected from peritoneal drainage tubes; CRC: colorectal cancer; CRT: chemo-radio therapy; CRP: C-reactive protein; AL: anastomotic leakage; LDH: lactate dehydrogenase; NLR: neutrophil-to-lymphocyte ratio.

**Table 4:** Subgroup- and sign-based recommendations for managing anastomotic leakage.

Subgroups	Sign *	AL Level	Recommend
POD 3–5 with CV 40–70	+	Low	Observe clinical symptoms, offer conservative treatment, and continue monitoring of CRP level.
POD 5–7 with CV 40–70	−	No	Discharge.
	+	Low	Delay discharge, offer conservative treatment, continue monitoring of CRP level, and observe clinical symptoms.
POD 3–5 with CV 70–150	−	No/Low	Observe clinical symptoms, offer conservative treatment, and continue monitoring of CRP level.
	+	Moderate	Swiftly rule out AL with contrast-enhanced CT.
POD 5–7 with CV 70–150	−	Moderate	Swiftly rule out AL with contrast-enhanced CT. If the CT does not confirm AL, consider delaying discharge.
	+	High	If CT confirms AL, proceed with surgery or continue conservative management.

Sign *: presence (+) or absence (−) of clinical signs, abnormal vital signs, or leukocytosis; POD: postoperative day; CV: cut-off value (mg/L); AL: anastomotic leakage; CRP: C-reactive protein; CT: computed tomography.

## Data Availability

The original contributions presented in this study are included in the article. Raw data supporting the conclusions of this article will be made available by the authors upon request. Inquiries can be directed to the corresponding author.
